# Vitamin C preferentially kills cancer stem cells in hepatocellular carcinoma via SVCT-2

**DOI:** 10.1038/s41698-017-0044-8

**Published:** 2018-01-08

**Authors:** Hongwei Lv, Changzheng Wang, Tian Fang, Ting Li, Guishuai Lv, Qin Han, Wen Yang, Hongyang Wang

**Affiliations:** 1 International Cooperation Laboratory on Signal Transduction, Eastern Hepatobiliary Surgery Institute, Second Military Medical University, 200438 Shanghai, China; 2 The Fifth Department of Hepatic Surgery, Eastern Hepatobiliary Surgery Hospital, Second Military Medical University, 200438 Shanghai, China; 3 National Center for Liver Cancer, 201805 Shanghai, China; 4 State Key Laboratory of Oncogenes and Related Genes, Shanghai Cancer Institute, Renji Hospital, Shanghai Jiaotong University, 200032 Shanghai, China

## Abstract

Vitamin C (L-ascorbic acid, ascorbate, VC) is a potential chemotherapeutic agent for cancer patients. However, the anti-tumor effects of pharmacologic VC on hepatocellular carcinoma (HCC) and liver cancer stem cells (CSCs) remain to be fully elucidated. Panels of human HCC cell lines as well as HCC patient-derived xenograft (PDX) models were employed to investigate the anti-tumor effects of pharmacologic VC. The use of VC and the risk of HCC recurrence were examined retrospectively in 613 HCC patients who received curative liver resection as their initial treatment. In vitro and in vivo experiments further demonstrated that clinically achievable concentrations of VC induced cell death in liver cancer cells and the response to VC was correlated with sodium-dependent vitamin C transporter 2 (SVCT-2) expressions. Mechanistically, VC uptake via SVCT-2 increased intracellular ROS, and subsequently caused DNA damage and ATP depletion, leading to cell cycle arrest and apoptosis. Most importantly, SVCT-2 was highly expressed in liver CSCs, which promoted their self-renewal and rendered them more sensitive to VC. In HCC cell lines xenograft models, as well as in PDX models, VC dramatically impaired tumor growth and eradicated liver CSCs. Finally, retrospective cohort study showed that intravenous VC use was linked to improved disease-free survival (DFS) in HCC patients (adjusted HR = 0.622, 95% CI 0.487 to 0.795, *p* < 0.001). Our data highlight that pharmacologic VC can effectively kill liver cancer cells and preferentially eradicate liver CSCs, which provide further evidence supporting VC as a novel therapeutic strategy for HCC treatment.

## Liver cancer: vitamin C preferentially kills cancer stem cells and improves patient outcomes

Pharmacologic doses of vitamin C preferentially eradicate liver cancer stem cells and are associated with improved outcomes in patients. A team led by Hong-Yang Wang and Wen Yang from the Second Military Medical University in Shanghai, China, showed that clinically achievable concentrations of vitamin C effectively killed liver cancer cells and preferentially eradicated cancer stem cells in culture and in mouse transplant models. Cells with higher expression levels of a vitamin C transporter protein were more susceptible to the treatment, which explains why cancer stem cells, which highly express this transportor and use it for their own self-renewal, were especially sensitive to take in vitamin C, which led to a cascade that resulted in DNA damage, energy depletion, and ultimately cell death. A retrospective analysis of 613 patients with liver cancer showed that those who received intravenous vitamin C lived longer without disease relapse.

## Introduction

Liver cancer is the sixth most frequent cancer and the second leading cause of cancer-related death worldwide.^1^ Hepatocellular carcinoma (HCC) accounts for over 80% of primary liver cancer cases and it is characterized by a high recurrence rate and heterogeneity.^2^ These pathological properties may flow from cancer stem cells (CSCs), which are capable of self-renewal and differentiation responsible for tumor progression, metastasis, and chemotherapy-resistance.^3^^,^^4^ Therefore, eradication of CSCs is emerging as a novel treatment strategy for liver cancer.

Vitamin C (L-ascorbic acid, ascorbate, VC), an important natural antioxidant, has a controversial history in cancer treatment. In the 1970s, Pauling and Cameron performed clinical trials showing efficacy of intravenous ascorbate in prolonging the survival of patients with terminal cancer.^5–7^ However, these researches were heavily criticized after subsequent double-blind and placebo-controlled trials using oral VC at the Mayo Clinic failing to show any benefit.^8^^,^^9^ It was recognized later that the route of VC administration was the key reason for the discrepancy. The originally reported studies using intravenous VC produces much higher plasma concentrations than the subsequent trials employing oral VC.^10^ More recently, Chen et al. have revealed that ascorbate at pharmacologic concentrations (0.3–20 mM) achieved only by intravenously (i.v.) administration selectively kills a variety of cancer cell lines in vitro, but has little cytotoxic effect on normal cells.^11–13^ Furthermore, high-dose parenteral VC administration represses the growth of numerous cancers in xenografts models including pancreatic cancer, ovarian cancer, prostate cancer, colon cancer, mesothelioma, breast cancer, and neuroblastoma.^13–16^ These observations have reactivated interest in anti-tumor effect of pharmacological VC globally. Yet, the detailed mechanisms underlying VC-induced cytotoxicity and the potential mechanisms modulating the differences in the sensitivity of cancer cells to VC are poorly understood. Additionally, whether VC has toxic effect on CSCs remains an open question.

Ascorbic acid (the reduced form of vitamin C) is specifically transported into cells by sodium-dependent vitamin C transporters (SVCTs).^17^ Two different isoforms of SVCTs, SVCT-1 (encoded by the *SLC23A1* gene) and SVCT-2 (*SLC23A2*), have been cloned.^18^ SVCT-1 is predominantly expressed in epithelial tissues, whereas the expression of SVCT-2 is ubiquitous.^19^ With respect to liver, SVCT-2 is the key protein responsible for VC uptake.^20^ SVCT-2 has higher affinity for VC than SVCT-1.^21^ Furthermore, genetic variations in SVCT-2 are closely associated with the risk of various cancers including gastric cancer, lymphoma, and head and neck squamous cell carcinomas.^22–24^ However, SVCT-2 expression and function in cancer and CSCs remain poorly characterized. So we hypothesize that SVCT-2 expression mainly responsible for VC uptake is linked to the differential susceptibility of liver cancer cells and CSCs to VC-induced cytotoxicity. Moreover, we investigate the mechanisms underlying VC-induced cell death and expression levels of SVCT-2 in HCC and CSCs.

## Results

### SVCT-2 is highly expressed in liver CSCs and is required for the maintenance of liver CSCs

**Fig. 1:**
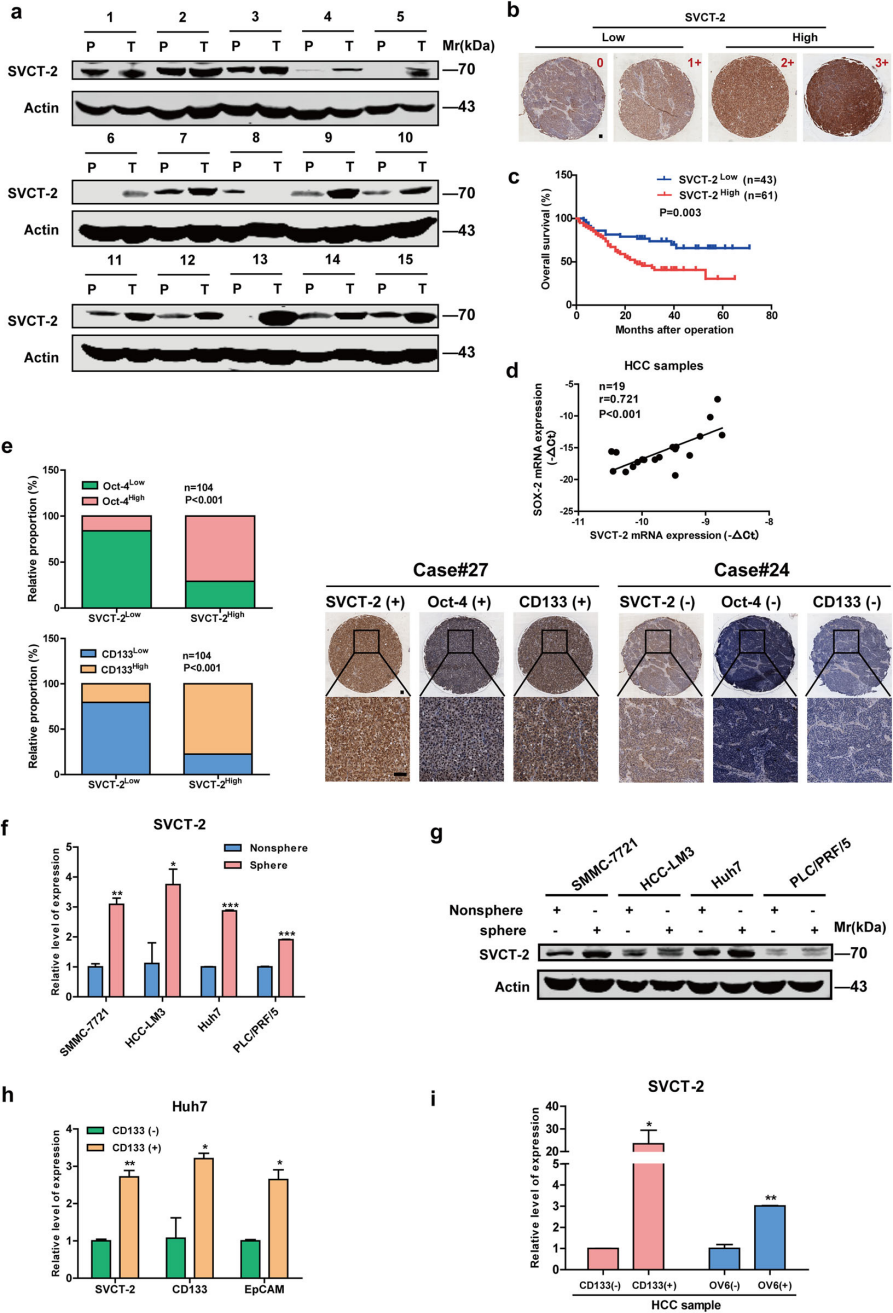
SVCT-2 is highly expressed in liver CSCs. **a** SVCT-2 expression was verified in HCC patient samples by immunoblotting. Samples derived from the same experiment and gels/blots were processed in parallel. **b** SVCT-2 immunohistochemistry staining in HCC tumor microarray (*n* = 104). Staining intensity grade was indicated in the upper right corner. Low SVCT-2 expression: grade 0/1+; high SVCT-2 expression: grade 2+/3+. Scale bars = 100 μm. **c** Kaplan–Meier analysis of overall survival in 104 HCC patients according to SVCT-2 expression. **d** SVCT-2 and Sox-2 expressions were detected by quantitative RT-PCR, followed by correlation analysis in HCC tissues. **e** Left: correlation analysis of SVCT-2 expressions with Oct-4 or CD133 expressions in HCC tissues. Right: IHC analysis of SVCT-2, Oct-4, and CD133 expressions in HCC tissues. Scale bars = 100 μm. **f**, **g** SVCT-2 is preferentially expressed in tumorspheres generated from HCC cells than nonspheres by qPCR (**f**) and immunoblotting (**g**). Samples derived from the same experiment and gels/blots were processed in parallel. **h**, **i** Relative expression of SVCT-2 was detected in CD133+ or OV6+ cell populations enriched from HCC cells (**h**) and HCC samples (**i**) in comparison to those of CD133− or OV6− cell subsets. P peri-tumor, T tumor

**Fig. 2:**
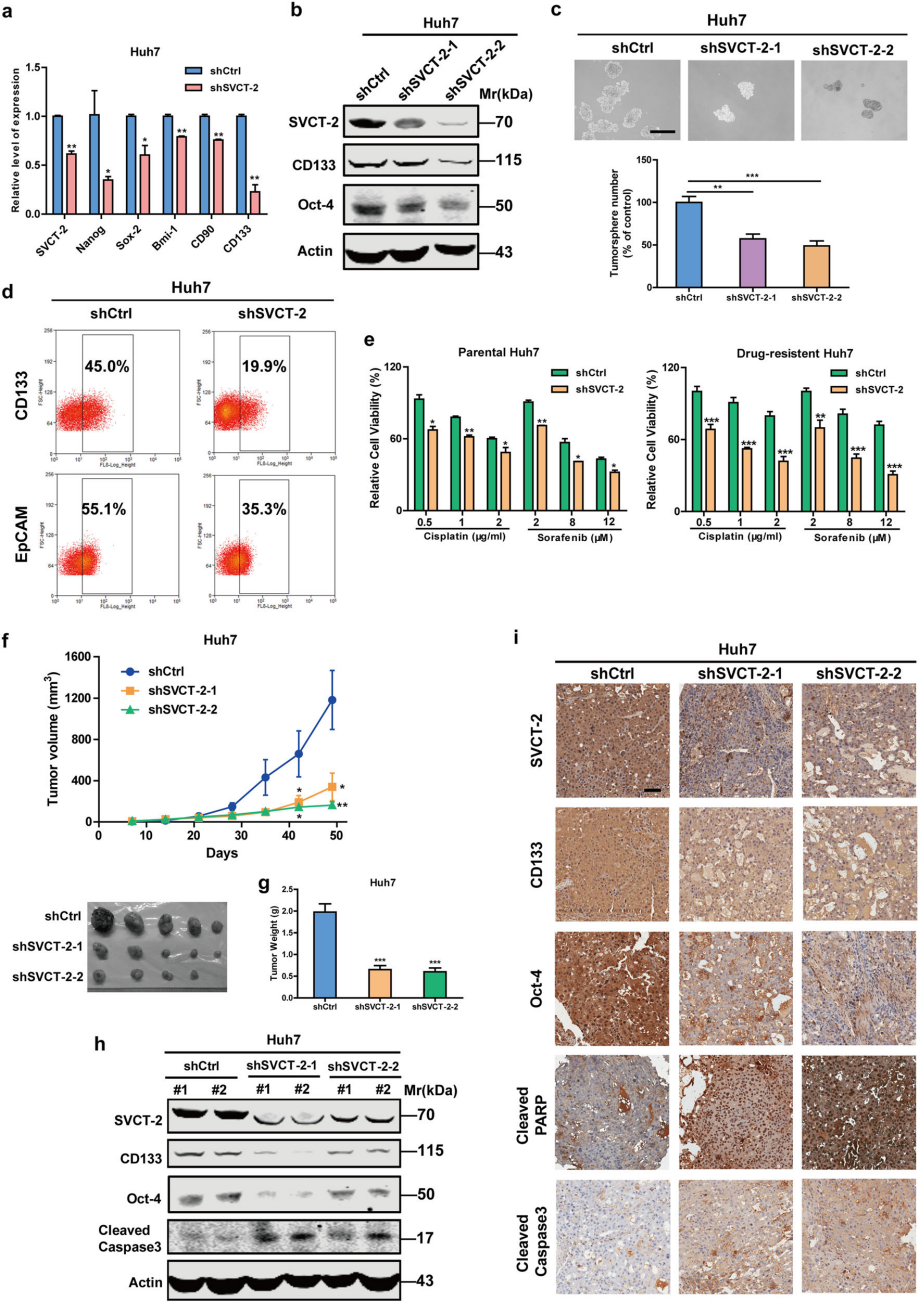
SVCT-2 is required for the maintenance of liver CSCs. **a** qRT-PCR analysis for stemness markers in shSVCT-2 cells and shCtrl cells. **b** Western blot analysis showing SVCT-2, CD133, and Oct-4 expressions in shSVCT-2 cells and shCtrl cells. Samples derived from the same experiment and gels/blots were processed in parallel. **c** shSVCT-2 cells and shCtrl cells were cultured for sphere-formation assays. Scale bars = 150 μm. **d** Flow cytometric analysis for the proportion of CD133+ or EpCAM+ cells in shSVCT-2 cells and shCtrl cells. **e** Left: shSVCT-2 and shCtrl parental Huh7 cells were treated with indicated concentrations of cisplatin and sorafenib for 48 h. Right: shSVCT-2 and shCtrl cisplatin-resistant or sorafenib-resistant Huh7 cells were treated with indicated concentrations of cisplatin and sorafenib for 48 h. Cell viability was determined by the CCK-8 assay. **f**, **g** shSVCT-2 and shCtrl cells (1 × 10^6^) were injected subcutaneously into nude mice. Tumor sizes were measured every week (**f**). After ~21 days of treatment, mice were euthanized and total tumor weights were measured (**g**). **h** Western blot analysis showing SVCT-2, CD133, Oct-4, and cleaved caspase 3 expressions in shSVCT-2 cells and shCtrl cells-derived tumor tissues from mice. Samples derived from the same experiment and gels/blots were processed in parallel. **i** IHC analysis showing SVCT-2, CD133, Oct-4, cleaved PARP, and cleaved caspase 3 expressions in shSVCT-2 cells and shCtrl cells-derived tumor tissues. Scale bars = 100 μm. Data are representative of at least three independent experiments and shown as mean ± s.d. (*p < 0.05; **p < 0.01; ***p < 0.001)

As illustrated in Fig. 1a, SVCT-2 was highly expressed in HCC samples in comparison to peri-tumor tissues. Furthermore, we employed tissue microarray immunohistochemistry to examine the prognostic significance of SVCT-2 expression in clinical tumor samples from cohorts of HCC patients (*n* = 104) (Fig. 1b). Importantly, high expression (grade 2+/3+) of SVCT-2 was in agreement with poorer overall survival (OS) of HCC patients (Fig. 1c) and more aggressive tumor behavior (Supplementary Table 1) compared to low or grade 0/1+ SVCT-2 expression. Intriguingly, SVCT-2 expression was positively correlated with stemness-related genes Sox-2, Oct-4, Lin28 or CSC marker CD133 (Fig. 1d, e). Sphere formation is well established to enrich CSCs on the basis of their self-renewing capacity.^3^ In vitro, we found that SVCT-2 expression was dramatically increased in the spheres derived from HCC cells compared with the corresponding adherent cells (Fig. 1f, g). Then, we isolated CD133+^25^ or OV6+^26^^,^^27^cell populations from both cultured HCC cell lines and HCC patient samples. Elevated expression of SVCT-2 was also detected in CD133+ or OV6+ cell populations than CD133− or OV6− cell subsets (Fig. 1h, i), suggesting that SVCT-2 is enriched in liver CSCs. To further determine the pathological role of SVCT-2 in liver CSCs, we knocked down SVCT-2 in Huh7 cells. SVCT-2 silencing dramatically declined expressions of stemness-related markers at both mRNA and protein levels (Fig. 2a, b). Additionally, sphere formation was markedly decreased in shSVCT-2 cells compared to shCtrl cells (Fig. 2c). Furthermore, knockdown of SVCT-2 significantly reduced the proportion of CD133+ or EpCAM+ cells (Fig. 2d) as well as the resistance to chemotherapeutic drugs in both parental and cisplatin-resistant or sorafenib-resistant Huh7 cells, which were established by continuous stepwise selection in increasing concentration of cisplatin or sorafenib from the parental cell lines over several months in our lab (Fig. 2e). In in vivo models, SVCT-2 deficiency remarkably decreased xenograft tumor growths and weights (Fig. 2f, g). Consistent with in vitro results, the expressions of stemness markers (CD133 and Oct-4) were reduced in shSVCT-2 cells-derived tumor tissues compared to shCtrl cells-derived tumor tissues from mice (Fig. 2h, i). Moreover, SVCT-2 deficiency promoted apoptotic markers (cleaved caspase 3 and cleaved poly(adenosine diphosphate–ribose) polymerase (PARP)) expressions in vivo (Fig. 2h, i). Altogether, these data suggest that SVCT-2 is preferentially expressed in liver CSCs and is required for the maintenance of liver CSCs.

### SVCT-2 determines the differential susceptibility to pharmacological VC-induced cell death

**Fig. 3:**
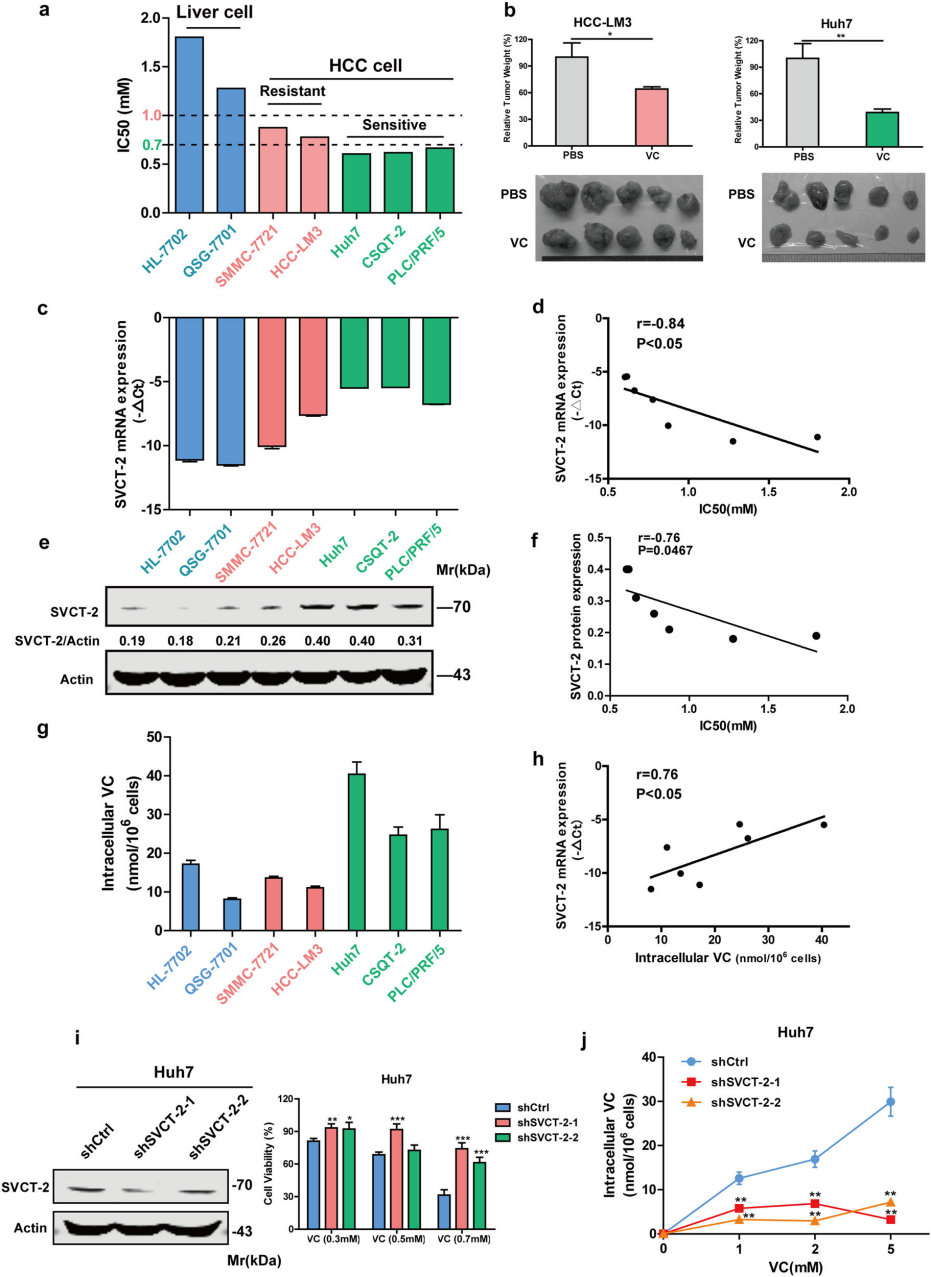
SVCT-2 determines the differential susceptibility to pharmacological VC-induced cell death. **a** IC50 values of VC in HCC cell lines and immortalized liver cell lines. These cells were treated with various concentrations of VC for 48 h. Cell viability was determined by the CCK-8 assay. **b** Relative weights of tumors from HCC-LM3 cells and Huh7 cells subcutaneously inoculated into nude mice after VC or PBS treatment. **c** SVCT-2 mRNA expressions in HCC cell lines and immortalized liver cell lines were detected by qRT-PCR. **d** Correlation between SVCT-2 mRNA expressions and IC50 values of VC in HCC cell lines and immortalized liver cell lines. **e** Western blot analysis showing expressions of SVCT-2 in HCC cell lines and relative normal liver cells. Actin served as a loading control. Samples derived from the same experiment and gels/blots were processed in parallel. **f** Correlation between SVCT-2 protein expression and IC50 values of VC in HCC cell lines and relative normal liver cells. **g** Intracellular VC concentration in the tested cells after exposure to 2 mM VC for 1 h. **h** Correlation between SVCT-2 mRNA expression and intracellular VC concentration in tested cells after VC treatment. **i** Left: Huh7 cells were transfected with SVCT-2-shRNA or scramble shRNA and the SVCT-2 expression was analyzed by immunoblotting. Samples derived from the same experiment and gels/blots were processed in parallel. Right: Huh7 cells transfected with SVCT-2-shRNA or scramble shRNA were treated with indicated doses of VC for 48 h. Cell viability was determined by the CCK-8 assay. **j** Intracellular VC concentrations in shSVCT-2 cells and shCtrl cells after treatment with VC at the indicated doses for 1 h. Data are representative of at least three independent experiments and shown as mean ± s.d. (*p < 0.05; **p < 0.01; ***p < 0.001)

As evidenced by clinical pharmacokinetics analyses,^10^ pharmacologic concentrations of plasma VC higher than 0.3 mM are achievable only from i.v. administration. To mimic potential clinical i.v. use, we treated five human HCC cell lines and two immortalized liver cell lines (HL-7702 and QSG-7701) with VC concentrations ranging from 0.3 to 1.5 mM. The viabilities of HCC cells were dramatically decreased after exposure to VC in dose-dependent manner, whereas the cytotoxicity of VC to immortalized liver cells was much weaker (Supplementary Fig. 1a, b). For all HCC cell lines, VC concentrations leading to 50% decrease in cell survival (IC50 values) were less than 1 mM, whereas IC50 values of VC in immortalized liver cell lines were obviously higher than 1 mM (Fig. 3a). These tested cells could be divided into three groups according to IC50 value of VC, the immortalized liver cells (HL-7702 and QSG-7701) with IC50 > 1 mM, VC-resistant HCC cells (SMMC-7721 and HCC-LM3) with 0.7 mM < IC50 < 1 mM, and VC-sensitive cells (Huh7, CSQT-2, and PLC/PRF/5) with IC50 < 0.7 mM (Fig. 3a). The inhibitory effect of VC was further confirmed in HCC-LM3 and Huh7 cell xenograft models in vivo. As shown in Fig. 3b, tumor derived from VC-sensitive Huh7 cells exhibited lower relative tumor weight compared with VC-resistant HCC-LM3 cells after VC treatment, in consistent with in vitro findings.

To investigate whether the difference in susceptibility to VC results from distinct concentrations of VC flow into cells, we initially examined the expressions of SVCT-1 and SVCT-2, both of which are responsible for VC uptake into cells, in tested cells. Interestingly, both the mRNA and protein levels of SVCT-2 were inversely correlated with IC50 values of VC in tested cells (Fig. 3c–f), whereas expressions of SVCT-1, which has lower affinity for VC than SVCT-2,^21^ were irrelevant to the IC50 values (Supplementary Fig. 1c, d). Moreover, SVCT-2 expression levels were positively correlated with intracellular VC concentrations in tested cells after VC treatment (Fig. 3g, h). To further explore the role of SVCT-2 in VC sensitivity, we knocked down SVCT-2 expression via short hair RNA (shRNA) on Huh7 cell line expressing high levels of SVCT-2 (Fig. 3i). Compared with control cells, the viabilities of shSVCT-2 cells significantly increased following VC treatment, implying resistance to VC (Fig. 3i and Supplementary Fig. 1e, f). Meanwhile, VC flow into shSVCT-2 cells dramatically decreased (Fig. 3j). These results indicate that differential sensitivity to VC may result from variations in VC flow into cells, which is dependent on SVCT-2 expression.

### Pharmacological VC preferentially kills liver CSCs in vitro

**Fig. 4:**
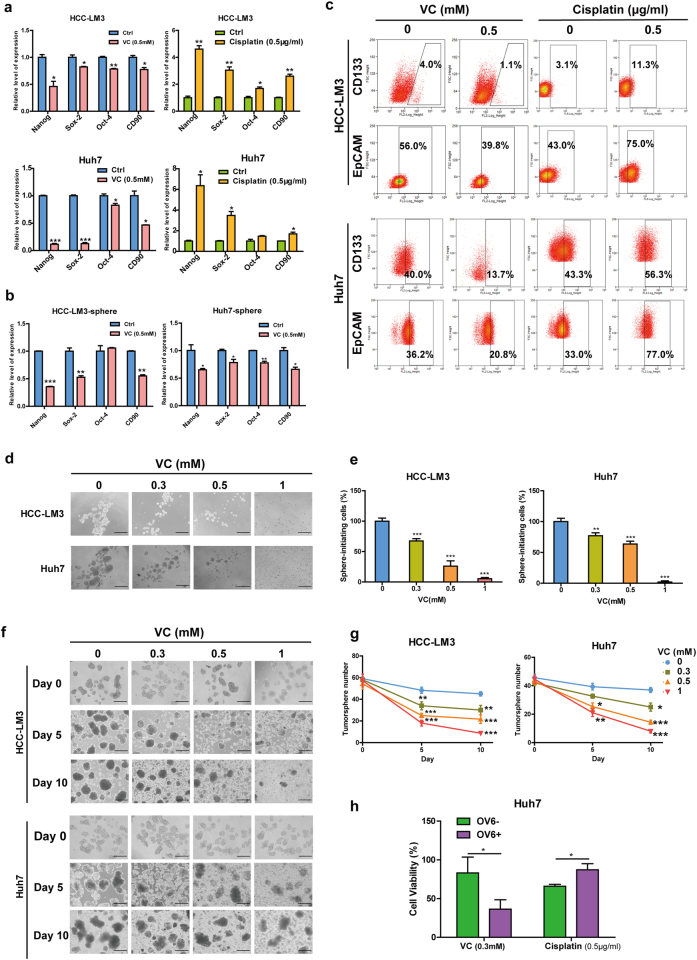
Pharmacological VC preferentially eradicates liver CSCs in vitro. **a** qRT-PCR analysis for stemness markers in the HCC cells untreated or treated with 0.5 mM VC or 0.5 μg/ml cisplatin for 48 h. **b** qRT-PCR analysis for stemness markers in tumorspheres derived from HCC cells untreated or treated with 0.5 mM VC for 48 h. **c** Flow cytometric analysis for the proportion of CD133+ or EpCAM+ cells in HCC cells untreated or treated with 0.5 mM VC or 0.5 μg/ml cisplatin for 48 h. **d**, **e** Representative images of the HCC cells cultured under non-adherent condition with VC at 0.3–1 mM or PBS (control) for 5 days (**d**). Quantification of tumorspheres in the same experiment (**e**). Scale bars = 150 μm. **f**, **g** Representative images of tumorspheres at day 5 of culture treated with the indicated concentrations of VC (**f**). Number of tumorspheres was counted every 5 days for 10 days (**g**). Scale bars = 150 μm. **h** OV6+ and OV6− cells obtained by magnetic sorting from Huh7 cells were treated with 0.5 mM VC or 0.5 μg/ml cisplatin for 48 h. Data are representative of at least three independent experiments and shown as mean ± s.d. (*p < 0.05; **p < 0.01; ***p < 0.001)

In light of above findings showing enrichment of SVCT-2 in liver CSCs, we next evaluated whether liver CSCs were more sensitive to VC-induced cell death. Intriguingly, in contrast to the effect of conventional chemotherapeutic agent cisplatin, to which CSCs are known to resist,^28^ VC treatment markedly downregulated the expressions of stemness-related genes and reduced the percentage of CD133+,^25^ EpCAM+,^29^ or OV6+^26^^,^^27^ CSCs both in HCC cells and tumorspheres (Fig. 4a–c, h). We further determined the effect of pharmacologic VC on liver CSCs self-renewal, as evidenced by the capacity of CSCs to form spheroids in vitro. As a result, high-dose VC significantly impaired both the tumorspheres initiation (Fig. 4d, e) and the growth of established tumorspheres derived from HCC cells (Fig. 4f, g) in a time-dependent and dose-dependent manner.

### SVCT-2-dependent mechanisms of pharmacological VC-induced cell death

**Fig. 5:**
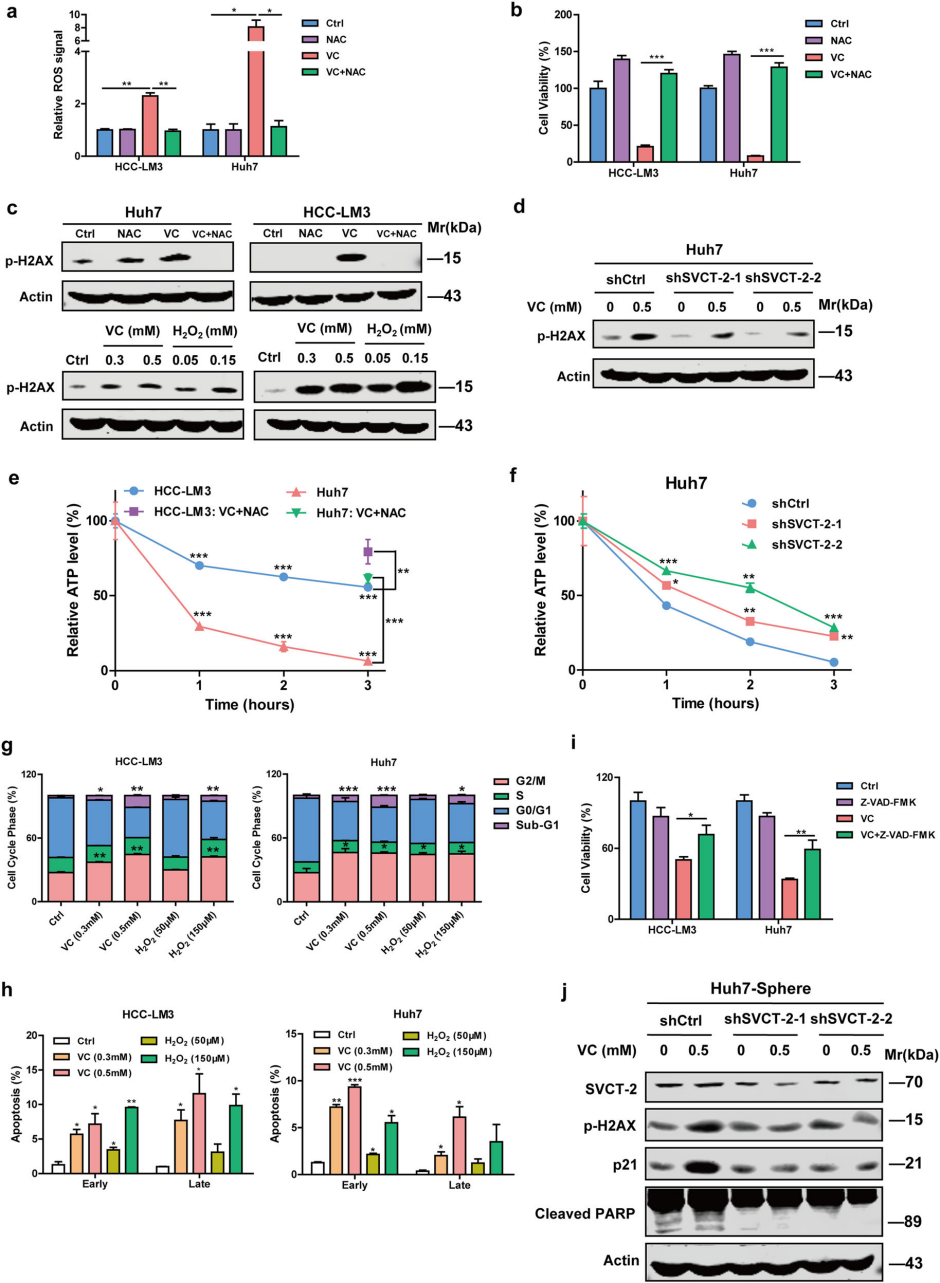
VC uptake via SVCT-2 increases intracellular ROS and subsequently causes DNA damage and ATP depletion, further leading to cell cycle arrest and apoptosis. **a** Quantification of ROS levels in HCC cells. Cells were treated with 2 mM VC for 1 h after pretreatment with 2 mM NAC. Then, the cells were incubated with DCF-DA for 30 min and analyzed by flow cytometer. **b** HCC cells were treated with 1 mM VC for 48 h after pretreatment with 2 mM NAC. Cell viability was determined by the CCK-8 assay. **c** Western blot analysis showing expressions of p-H2AX in HCC cells exposed to 0.5 mM VC with or without NAC for 48 h and p-H2AX induced by VC or H_2_O_2_ in a dose-dependent manner. Samples derived from the same experiment and gels/blots were processed in parallel. **d** Western blot analysis showing p-H2AX in shSVCT-2 cells and shCtrl cells treated with VC for 48 h. Samples derived from the same experiment and gels/blots were processed in parallel. **e** ATP levels of HCC cells at different time points after treating with 2 mM VC. At 3 h, addition of NAC before VC reversed the declines of ATP levels induced by VC treatment in HCC-LM3 cells (purple square) and Huh7 cells (green triangle). **f** ATP levels of shSVCT-2 cells and shCtrl cells at different time points after VC treatment. ATP was normalized to the total cellular protein in each sample. **g** Flow cytometric quantification of cell cycle phase of HCC cells treated with VC or H_2_O_2_ for 48 h. **h** Flow cytometric quantification o Annexin V-FITC/PI double-staining of HCC cells treated with VC or H_2_O_2_ for 48 h. Early apoptosis: Annexin V positive and PI negative; late apoptosis: both Annexin V and PI positive. **i** HCC cells were treated with 1 mM VC for 48 h after pretreatment with the pan-caspase inhibitor Z-VAD-FMK for 1 h. Cell viability was determined by the CCK-8 assay. **j** Western blot analysis showing SVCT-2, p-H2AX, p21, and cleaved PARP expression in shSVCT-2 tumorspheres and shCtrl tumorspheres treated with VC for 48 h. Samples derived from the same experiment and gels/blots were processed in parallel. Data are representative of at least three independent experiments and shown as mean ± s.d. (*p < 0.05; **p < 0.01; ***p < 0.001)

Intracellular reactive oxygen species (ROS) levels increased in two HCC cells differentially expressing SVCT-2 protein after exposure to VC. More ROS was detected in Huh7 cells with relative higher SVCT-2 expression compared to HCC-LM3 cells (Fig. 5a). The antioxidant, N-acetyl-L-cysteine (NAC), preventing VC-induced ROS production (a ROS scavenger), completely restored the viability and colony formation among VC-treated cells (Fig. 5b and Supplementary Fig. 2a). Furthermore, DNA double-strand damage was found following VC treatment, as shown by phosphorylation of histone 2AX (H2AX) depending on VC concentration. DNA damage was prevented by NAC and H_2_O_2_ (a major form of ROS) induced similar effects (Fig. 5c). Additionally, SVCT-2 knockdown markedly reduced expression of phosphorylated H2AX (p-H2AX) induced by VC, suggesting VC-induced DNA damage is dependent on SVCT-2 (Fig. 5d). A PARP inhibitor, Olaparib, inhibiting DNA repair and enhancing DNA damage, significantly increased VC-induced cell death (Supplementary Fig. 2b, c). Addition of cisplatin, a conventional chemotherapeutic regimen, to VC enhanced DNA damage (Supplementary Fig. 2d) and exhibited an synergistic effect on cell death in comparison to either drug alone, as evidenced by combination index (CI), which was calculated with isobologram principles^30^ to determine synergism (CI < 1), additive effect (CI = 1), or antagonism (CI > 1) (Supplementary Fig. 2d).

It is well established that excessive oxidative stress causes depletion of cellular adenosine triphosphate (ATP).^31^ ATP decreases dependent on time were observed in VC-treated HCC cells and reduction in ATP levels was greater in Huh7 cells expressing higher SVCT-2 than HCC-LM3 cells (Fig. 5e). NAC dramatically reversed VC-induced ATP depletion in HCC cells, suggesting the necessity of ROS in reducing ATP levels (Fig. 5e). Similarly, SVCT-2 silencing also suppressed the depletion of ATP in Huh7 cells following VC treatment in different time points (Fig. 5f). Furthermore, VC induced G2/M phase cell cycle arrest, accompanied by significant reduce in G0/G1 phases and enhanced expression of cyclin-dependent kinase inhibitor p21 in a concentration-dependent manner, consistent with findings with H_2_O_2_ and VC-triggered cell cycle arrest was inhibited in the presence of NAC (Fig. 5g and Supplementary Fig. 2f). Knockdown of SVCT-2 remarkably repressed the activation of p21 induced by VC (Supplementary Fig. 2g). Additionally, a characteristic hypodiploid DNA content peak (sub-G1) representing apoptotic cells was detected, indicating VC-induced apoptosis after G2/M arrest (Fig. 5g). Indeed, the proportions of early and late apoptotic cells were significantly increased in a VC concentration-dependent manner (Fig. 5h). Caspase 3 and PARP were cleaved in VC-treated cells (Supplementary Fig. 2f) and the VC-induced decrease in cell viability was partially recovered after pretreatment with Z-VAD-FMK, a pan-caspase inhibitor, implying that VC triggers caspase-dependent death in HCC cells (Fig. 5i and Supplementary Fig. 2e). Cleaved caspase 3 and PARP induced by VC were dramatically reduced in shSVCT-2 cells compared to shCtrl cells, suggesting that VC partially induces caspase-dependent apoptosis in SVCT-2-dependent manner (Supplementary Fig. 2g). Similarly, knocking down SVCT-2 markedly reversed the enhanced expressions of p-H2AX, p21, and cleaved-PARP induced by VC in tumorspheres (Fig. 5j).

We also tested whether VC-induced HCC cell death was dependent on autophagy.^32–34^ The cellular autophagy markers Beclin-1 and LC3B-II proteins were upregulated in VC-treated cells and addition of NAC suppressed expressions of these proteins (Supplementary Fig. 3a), implying that VC is involved in autophagy induction. However, inhibition of autography via an autophagy inhibitor (3-MA) (Supplementary Fig. 3b) or Beclin-1 knockdown had no effect on VC-induced cell death (Supplementary Fig. 3c, d). Therefore, VC triggers autophagy-independent death in human HCC cells. In addition to autophagy and apoptosis, necrosis is another major type of cell death and also functions as an alternative mode of programmed cell death.^35^^,^^36^ To test whether VC induces programmed necrosis or necroptosis, two small compound inhibitors necrostatin-1 (Nec-1) and necrosulfonamide (NSA) were employed to block the activity of central regulators in the programmed necrosis or necroptosis. As a result, neither of the inhibitors alleviated VC-induced cytotoxicity (Supplementary Fig. 3e, f). These results indicate that necroptosis may not be one of the cell death mechanisms triggered by VC. Altogether, these data indicate that VC influx into cells via SVCT-2 and increases intracellular ROS levels, which subsequently induces DNA damage and ATP depletion, leading to cell death partially via cell cycle arrest and caspase-dependent apoptosis, but not autophagy or necroptosis.

### Pharmacological VC impairs tumor growth and eradicates liver CSCs in vivo

**Fig. 6:**
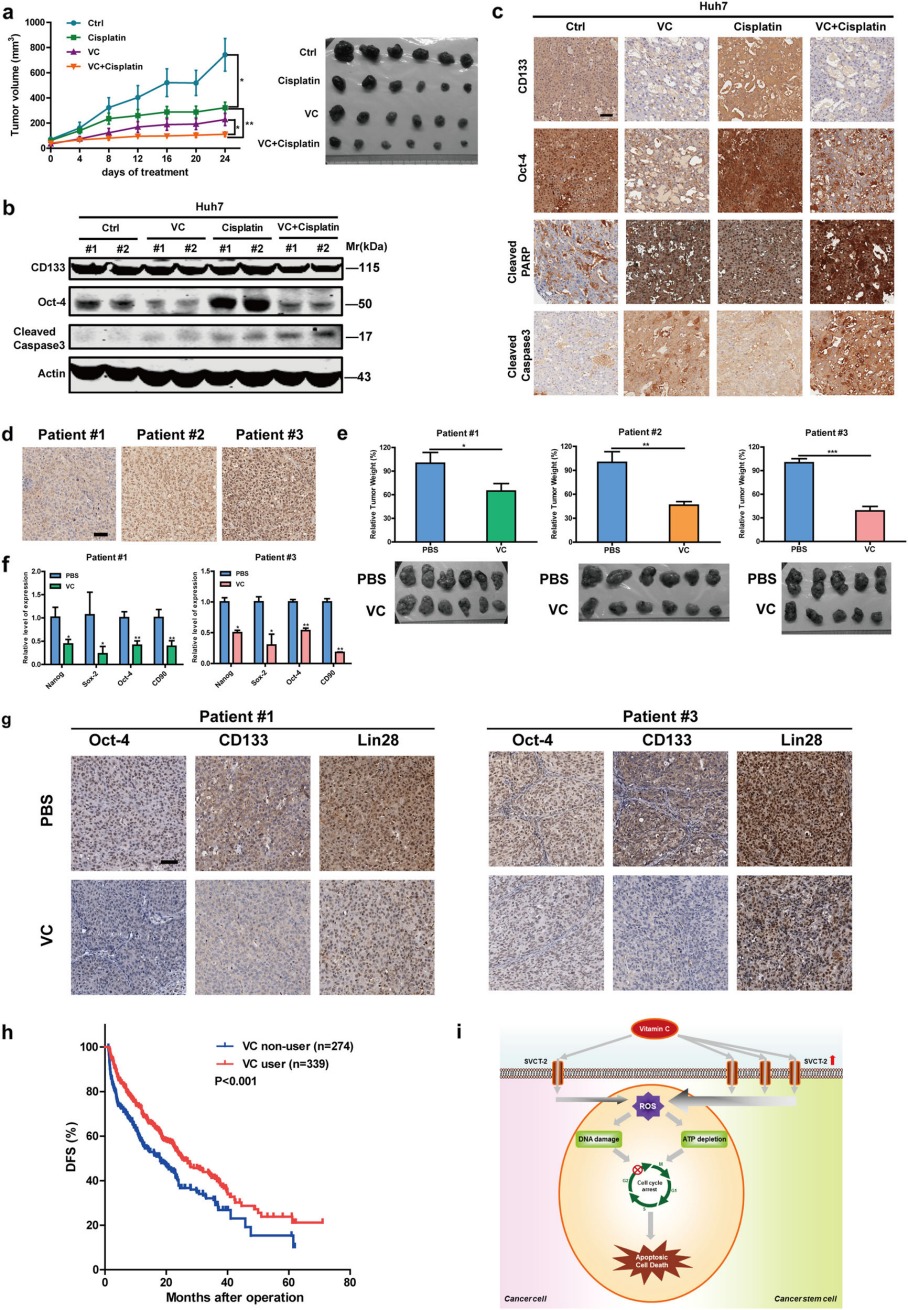
Pharmacological VC impairs tumor growth and preferentially kills liver CSCs in vivo, and intravenous VC reduces the risk of post-surgical HCC progression. **a** Huh7 cells were subcutaneously inoculated into nude mice. When tumors grew to ~50 mm^3^, treatment commenced with intraperitoneal injection of VC (4 g/kg, twice every day) and cisplatin (Cp; 3 mg/kg, twice per week) either alone or in combination. Tumor sizes were measured twice per week. After ~21 days of treatment, mice were euthanized and total tumor weights were measured. **b** Western blot analysis showing stemness and apoptotic markers expressions in tumor xenograft after treatment of VC and cisplatin either alone or in combination. Samples derived from the same experiment and gels/blots were processed in parallel. **c** IHC analysis showing stemness and apoptotic markers expressions in tumor xenograft after treatment of VC and cisplatin either alone or in combination. Scale bars = 100 μm. **d** IHC analysis showing SVCT-2 expression between PDXs from patient #1, #2, and #3. Scale bars = 100 μm. **e** Relative weights of PDXs from patient #1, #2, and #3 after ~21 days of VC treatment. PDXs were treated intraperitoneally twice daily with either VC (4.0 g/kg) or vehicle (PBS). **f** qRT-PCR analysis for stemness markers in PDXs from patient #1 and #3 after treatment of either VC or vehicle (PBS). **g** IHC analysis showing Oct-4, CD133, and Lin28 expressions in PDXs from patient #1 and #3 after treatment of either VC or vehicle (PBS). Scale bars = 100 μm. **h** DFS of 613 patients with primary HCC after initial hepatectomy receiving 2 g intravenous VC, or not. **i** Schematic showing how VC kills cancer cells and preferentially kills CSCs via SVCT-2. Data are representative of at least three independent experiments and shown as mean ± s.d. (*p < 0.05; **p < 0.01; ***p < 0.001)

To further confirm above findings in vivo, we established both HCC cell xenografts and HCC patient-derived xenografts (PDXs) models. Consistent with the in vitro results, stemness-related genes expressions in tumor xenograft were remarkably reduced after VC or VC+cisplatin treatment, whereas conventional cisplatin therapy alone led to the increase of CSCs (Fig. 6b, c). Interestingly, the combination of VC and cisplatin was even more effective in reducing tumor growth and weight (Fig. 6a). Furthermore, either VC or cisplatin alone resulted in increased apoptotic markers expressions, whereas VC and cisplatin combination further caused cell apoptosis in tumor xenograft (Fig. 6b, c). In HCC PDXs models with relative low and high SVCT-2 expression, VC treatment significantly delayed tumor growth (Fig. 6d, e). Intriguingly, PDX#2 and PDX#3, which had relative higher SVCT-2 expression, exhibited lower relative tumor growth and mass compared with PDX#1, suggesting hyper-sensitivity toward VC treatment (Fig. 6d, e). These results verify that VC inhibits tumor growth in HCC PDX models and SVCT-2 expression level is associated with VC response. Furthermore, qPCR and IHC analysis demonstrated that expression levels of CSC-associated genes and percentages of CSCs in PDXs dramatically declined after VC treatment, confirming the inhibitory role of VC in liver CSCs (Fig. 6f, g).

### Intravenous VC reduces the risk of post-surgical HCC progression

Liver protection treatment is regularly given to HCC patients after hepatectomy. VC is one of the numerous common hepatoprotectants.^37^ In our Eastern Hepatobiliary Surgery Hospital, Shanghai, China, some HCC patients received intravenous VC after hepatectomy. Pharmacokinetics studies in human show that 2 g of intravenous VC achieves a plasma concentration of nearly 1.5 mM.^10^ Interestingly, at extracellular concentrations greater than 1 mM, VC induces strong cytotoxicity to cancer cells including liver cancer cells, as demonstrated in the above studies.^38^ Therefore, we hypothesized that intravenous VC might reduce the risk of recurrence in HCC patients after curative liver resection.

Six hundred thirteen HCC patients who received curative liver resection as their initial treatment between 2008 and 2009 and met the inclusion criteria were enrolled in the analyses. HCC patients were divided into two groups: VC users and non-VC users. Three hundred thirty-nine participants (55.3%) received 2 g intravenous VC for 4 or more days after initial hepatectomy. As shown in Supplementary Table 2, the distribution of clinicopathologic factors between VC users and non-users was no significant difference. Intriguingly, the 5-year disease-free survival (DFS) for patients who received intravenous VC was 24%, as opposed to 15% for no intravenous VC-treated patients (*p* < 0.001) (Fig. 6h). Median DFS time for VC users was 25.2 vs. 18 months for VC non-users (*p* < 0.001). Univariate analysis revealed that tumor size ≥5 cm, multiple tumor numbers, AFP ≥ 20 μg/L, AFP ≥ 400 μg/L, tumor thrombus, and no post-surgical intravenous VC administration were significantly associated with shorter DFS (Table 1). Multivariate analysis further demonstrated that intravenous VC administration was an independent factor for improved DFS (adjusted HR = 0.622, 95% CI 0.487 to 0.795, *p* < 0.001) (Table 1). These results suggest that intravenous VC use is linked to improved DFS in HCC patients.

**Table 1:** Univariate/multivariate analysis of prognostic factors associated with the DFS of 613 HCC patients

	Univariate analysis		Multivariate analysis	
	HR (95% CI)	*p-*vaule	HR (95% CI)	*p-*vaule
VC use (user vs. non-user)	0.702 (0.572–0.861)	<0.001*	0.622 (0.487–0.795)	<0.001*
Age (≥60 vs. <60)	1.052 (0.835–1.326)	0.667		
Gender (male vs. female)	1.193 (0.886–1.608)	0.245		
HBV infection	1.043 (0.794–1.370)	0.763		
Tumor size (≥5 cm vs. <5 cm)	1.807 (1.466–2.229)	<0.001*	1.530 (1.181–1.982)	<0.001*
Tumor number (multiple vs. solitary)	2.086 (1.617–2.692)	<0.001*	1.774 (1.321–2.383)	<0.001*
AFP (μg/L)				
≥20 vs. <20	1.433 (1.162–1.766)	<0.001*	1.358 (1.054–1.750)	0.018*
≥400 vs. <400	1.412 (1.105–1.804)	0.006*		
Tumor differentiation (III–IV vs. I–II)	0.937 (0.764–1.150)	0.535		
Liver cirrhosis	1.112 (0.900–1.375)	0.325		
Microscopic tumor thrombus (present vs. absent)	1.372 (1.114–1.690)	0.003*		
Macroscopic tumor thrombus (present vs. absent)	2.518 (1.891–3.352)	<0.001*	1.682 (1.181–2.397)	0.004*
Tumor encapsulation (incomplete vs. complete)	1.015 (0.828–1.244)	0.888		

*p < 0.05

## Discussion

Despite the recent advances in liver cancer therapy, it remains one of the most lethal malignancies. VC has a controversial history in cancer treatment. In the 1970s, Pauling and Cameron reported that intravenous VC (10 g/day) was effective in prolonging the survival of cancer patients.^5–7^ However, clinical trials performed by Mayo Clinic found the same dose of VC ineffective in treating cancer by using it orally.^8^^,^^9^ It was recognized later that the route of VC administration was the main reason for the discrepancy. Pharmacologic concentrations of plasma VC, which are achievable only from i.v. administration other than oral VC, can kill cancer cells.^10^ Currently, pharmacologic VC has garnered increased interest in the field of cancer therapy. However, few studies have investigated the effect of VC on CSCs, the subpopulation responsible for tumor initiation, metastasis, recurrence, and resistance to chemotherapy.^3^^,^^4^ In this study, based on the elevated expression of SVCT-2, which is responsible for VC uptake, in liver CSCs, we revealed that clinically achievable concentrations of VC preferentially eradicated liver CSCs in vitro and in vivo. Additionally, we found that intravenous VC reduced the risk of post-surgical HCC progression in a retrospective cohort study.

As the key protein responsible for VC uptake in the liver, SVCT-2 played crucial roles in regulating the sensitivity to ascorbate-induced cytotoxicity.^34^ In this study, we also revealed that SVCT-2 expressions were inversely associated with IC50 values of VC and positively correlated with intracellular VC concentrations in HCC cells after VC treatment. Conversely, SVCT-2 silencing in Huh7 cells dramatically decreased the sensitivity to VC. Strikingly, we also observed that SVCT-2 was highly expressed in human HCC samples and preferentially elevated in liver CSCs. Knocking down SVCT-2 expression significantly affected self-renewal, chemoresistance, and tumorigenicity of liver CSCs. In this regard, SVCT-2 might serve as a potential CSC marker and therapeutic target in HCC. Unexpectedly, physiological concentration of VC does not markedly promote HCC in vitro. We found that low dose (0.1 mM) of VC had no significant influence on HCC cells growth and the stemness-related genes expressions (Supplementary Fig. 4a, b). Nevertheless, our in vitro conditions are unable to sufficiently mimic the in vivo environment with hypoxia, hypoglycemia, and other metabolic changes. Therefore, further studies are needed to evaluate the effect of physiological VC on HCC in vitro and in vivo.

CSCs play critical roles in regulating tumor initiation, relapse, and chemoresistance.^3^^,^^4^ In HCC, we have previously demonstrated that OV6+ liver CSCs exhibit resistance to chemotherapy and contribute to HCC progression and invasion.^26^^,^^27^ Contrary to expectations, VC is distinguished from other well-defined chemotherapeutic drug (e.g., cisplatin, doxorubicin) and VC treatment does not lead to the enrichment of CSCs. Instead, by detecting key features of CSCs in vitro and in vivo, we revealed that VC treatment dramatically reduced the self-renewal ability, expression levels of CSC-associated genes, and percentages of CSCs in HCC, indicating that CSCs were more susceptible to VC-induced cell death. Thus, as a drug for eradicating CSCs, VC may represent a promising strategy for treatment of HCC, alone or particularly in combination with chemotherapeutic drugs.

It is accepted that the cytotoxicity of pharmacologic VC is mediated by generation of sustainable ascorbate radical and H_2_O_2_.^11^ However, there is no general molecular mechanism suitable for heterogeneous cancer cells because H_2_O_2_ could produce downstream ROS and influence various cellular and molecular targets. Previous studies have reported multiple mechanisms in different cancers, including caspase-dependent and caspase-independent apoptosis,^39^ nonapoptotic cell death,^11^^,^^40^ autophagy, ^16^ autoschizis,^41^ ATP depletion,^25^ DNA damage,^25^^,^^42^ and cell cycle arrest.^42^ In HCC, we found that VC-generated ROS caused genotoxic stress (DNA damage) and metabolic stress (ATP depletion), which further activated the cyclin-dependent kinase inhibitor p21, leading to G2/M phase cell cycle arrest and caspase-dependent apoptosis in HCC cells (Fig. 6i). Furthermore, we demonstrated a synergistic effect of VC and chemotherapeutic drug cisplatin on killing HCC both in vitro and in vivo. It is known that cisplatin treatment also results in DNA damage despite through a distinct mechanism from that in VC.^43^ Cisplatin induces DNA damage via reaction of the platinum molecule with nucleophilic sites rather than ROS.^43^ As a result, VC and cisplatin combination led to larger extent of DNA damage in HCC cells than either use alone. Intravenous VC has also been reported to reduce chemotherapy-associated toxicity of carboplatin and paclitaxel in patients,^38^ but the specific mechanism needs further investigation.

Pharmacokinetics studies show that 2 g of intravenous VC achieves a plasma concentration of nearly 1.5 mM,^10^ a concentration sufficient to induce death in HCC cells, as evidenced by our in vitro studies. Our retrospective cohort study also showed that intravenous VC use (2 g) was related to the improved DFS in HCC patients after initial hepatectomy. In fact, several clinical trials of high-dose intravenous VC have been conducted in patients with advanced cancer and have revealed improved quality of life and prolonged OS.^44^ Considering the much higher dose (≥50 g) employed in these clinical trials, additional clinical trials will be needed to prove the safety, efficacy, and doses of VC in HCC treatment. All xenografts were performed in nude mice with compromised immune system to test the anti-tumor effect of VC in the above studies. Since VC may help boost body immune system to fight against cancer, we further examined the effect of high-dose VC on HCC progression and immune cells using normal mice. Similarly, VC treatment significantly inhibited growths of tumors derived from mouse liver cancer cells (Hepa1-6) in C57BL/6 mouse (Supplementary Fig. 4c, d). Furthermore, high-dose VC was not toxic to immune cells and major immune cell subpopulations in vivo (Supplementary Fig. 4e, f). Thus, the inhibitory effect of pharmacologic VC on liver cancer may be not mainly through the promotion of immune system. Taken together, our findings unravel the potential application of VC for HCC therapy. The mechanisms about how pharmacologic VC kills cancer cells and preferentially kills CSCs via SVCT-2 are summarized in Fig. 6i. Notably, we also propose that SVCT-2 is a new CSC marker and therapeutic target in HCC and its expression level may serve as a biomarker for VC response.

## Methods

### Patients

In the retrospective study, a total of 669 patients with primary HCC who underwent initial curative liver resection in the Eastern Hepatobiliary Surgery Hospital, Shanghai, China, from 2008 to 2009 were collected. Of these, 613 patients who met the inclusion criteria were finally enrolled. The inclusion criteria included: (1) the diagnosis of HCC was based on World Health Organization criteria; (2) none of the patients received chemotherapy or radiotherapy before the surgery. HCC patients were divided into two groups: VC users (*n* = 339) and VC non-users (*n* = 274). Patients who received 2 g intravenous VC for 4 or more days after initial hepatectomy were defined as VC users. The clinicopathological features of 613 patients were summarized in Supplementary Table 2. Additionally, a tissue microarray composed of HCC samples from 104 patients used to examine the prognostic significance of SVCT-2 expression (Fig. 1b, c) was obtained from the Eastern Hepatobiliary Surgery Hospital. The clinicopathological features of 104 patients were summarized in Supplementary Table 1. Another 19 fresh HCC tissues were also obtained from the Eastern Hepatobiliary Surgery Hospital to evaluate the correlation between SVCT-2 and stemness-related genes by qRT-PCR analysis (Fig. 1d). Patient consent was obtained prior to the start of the study. All studies were approved by the Ethical Committee of the Second Military Medical University (SMMU) and performed in accordance with relevant regulations and guidelines.

### In vivo xenograft assay

1 × 10^6^ shCtrl, shSVCT-2-1, and shSVCT-2-2 Huh7 cells were injected subcutaneously into the right flank of each male nude mouse (Chinese Science Academy, Shanghai, China). To investigate the role of VC in cancer treatment in vivo, 1 × 10^6^ human HCC cell lines (HCC-LM3 and Huh7) were injected subcutaneously into the right flank of each nude mouse and 1 × 10^6^ mouse liver cancer cell line (Hepa1-6) was injected subcutaneously into the right flank of each male C57BL/6 mouse (Chinese Science Academy, Shanghai, China). When tumors grew to ~50 mm^3^, mice were randomized into two groups (*n* = 6) and treatment commenced with intraperitoneal injection of 4 g/kg VC (equivalent to ~1.3 g/kg i.v.),^12^ a dose widely used in numerous studies to test the effect pharmacological VC on various cancer treatment in mouse model,^13,16,38^ or vehicle (PBS) twice every day for ~21 days. In another study, 1 × 10^6^ Huh7 cells were injected subcutaneously into the right flank of each nude mouse. When tumor volume had reached ~50 mm^3^, mice were randomized into four groups (*n* = 6) and treatment commenced with intraperitoneal injection as follows: (i) Ctrl, PBS twice daily; (ii) VC, vitamin C at 4 g/kg twice daily; (iii) Cp, cisplatin at 3 mg/kg twice per week; (iv) VC+Cp.

For HCC PDX model, fresh tumor specimens were procured from previously established PDX models (passage 2–3) and cut into small tissue blocks (~50 mm^3^) before engrafted subcutaneously into male nude mice (Chinese Science Academy, Shanghai, China). After 2–3 weeks, PDXs from patient #1 (*n* = 6) and patient #2 (*n* = 6) were intraperitoneally treated with either VC (4 g/kg) or vehicle (PBS) twice daily. Tumor size (length × width^2^ × 0.5) was measured twice per week after treatment. At ~25 days, all mice were euthanized and tumors were excised and weighed. Mice were employed between 4 and 6 weeks of age and the number of mice per group was selected to provide sufficient statistical power to the experiment based on the expected biological variation. Investigators were not blinded as to group allocation. All animal experiments were approved by the Ethical Committee of the SMMU and performed in accordance with relevant regulations and guidelines.

### Statistics

Statistical analysis was carried out using SPSS 22.0 software (SPSS Inc., USA). The data are presented as the mean ± s.d. Two-tailed Student’s *t*-test was used to determine the significance of differences between groups. Pearson’s correlation analysis was applied to determine the correlation between two variables. The survival rate was calculated using the Kaplan–Meier method and univariate survival analysis was done by the log-rank test. Multivariate analysis was performed using the Cox proportional hazards model. *p*-value < 0.05 was considered as significant.

Additional methods are described in Supplementary Information.

### Data availability

All data supporting the findings of this study are available within the paper and its Supplementary Information files.

## Electronic supplementary material

Supplementary Information

## Electronic supplementary material

**Supplementary information** accompanies the paper on the *npj Precision Oncology* website (10.1038/s41698-017-0044-8).
