# Supplementary material for: Vitamin C preferentially kills cancer stem cells in hepatocellular carcinoma via SVCT-2
Source: NPJ Precis Oncol. 2018 Jan 8;2:1. doi: 10.1038/s41698-017-0044-8 (PMC5871898; doi:10.1038/s41698-017-0044-8)
Supplement: Supplementary file 1 — Supplementary Information [file 41698_2017_44_MOESM1_ESM.doc]

**Supplementary Information**

**Vitamin C preferentially kills cancer stem cells in hepatocellular carcinoma via SVCT-2**

Hongwei Lv^1^*, Changzheng Wang^1,3^*, Tian Fang^1^*, Ting Li^1^, Guishuai Lv^1,2^, Qin Han^1^, Wen Yang^1,2#^, Hongyang Wang^1,2,4#^

^1^International Co-operation Laboratory on Signal Transduction, Eastern Hepatobiliary Surgery Institute, Second Military Medical University, Shanghai 200438, China;

^2^National Center for Liver Cancer, Shanghai 201805, China;

^3^The Fifth Department of Hepatic Surgery, Eastern Hepatobiliary Surgery Hospital, Second Military Medical University, Shanghai 200438, China;

^4^State Key Laboratory of Oncogenes and Related Genes, Shanghai Cancer Institute, Renji Hospital, Shanghai Jiaotong University, Shanghai 200032, China.

*These authors contributed equally to this work.

^#^Corresponding author

Correspondence:

Professor Hong-Yang Wang, hywangk@vip.sina.com

Professor Wen Yang, woodeasy66@hotmail.com

International Cooperation Laboratory on Signal Transduction, Eastern Hepatobiliary Surgery Institute/Hospital, 225 Changhai Road, Shanghai 200438, China.

Phone: 86-21-81875361; Fax: 86-21-65566851;

**Supplementary Methods**

**Cell culture and reagents**

The cell lines HL-7702, QSG-7701, SMMC-7721, HCC-LM3, Huh7, CSQT-2, and PLC/PRF/5 were purchased from Cell Bank of Type Culture Collection of Chinese Academy of Sciences (China). Cell lines were authenticated by STR profiling and verified to be mycoplasma negative. All cells were cultured in Dulbecco’s modified Eagle’s medium (DMEM) (Gibco, USA) supplemented with 10% fetal bovine serum, 100 units/ml penicillin, and 100 μg/ml streptomycin at 37°C in a humidified incubator with 5% CO_2_. L-ascorbate, NAC, 3-MA, and cisplatin were purchased from Sigma (USA). Z-VAD-FMK, olaparib, and Nec-1 were purchased from Selleck (USA).

**Cell viability and colony formation assay**

Cell viability was detected by Cell Counting Kit-8 (CCK-8) assay (Dojindo Laboratories, Japan) using 96-well plates. Seeded cell density was 4,000 per well. Cells were washed and incubated with CCK-8 at 37°C for 1 h. Then, the absorbance at 450 nm was determined with a Synergy 2 micro-plate Reader.

For colony formation assay, cells were seeded in a 6-well plate at 1,000 per well. Cells were treated with VC at the indicated doses for 24 h after pretreatement with NAC or 3-MA and then cultured for 2 weeks. Colonies were fixed with 4% paraformaldehyde and then stained with a crystal violet solution. Colony numbers were counted manually and representative views were pictured.

**qRT-PCR**

Cell and tissue total RNA was extracted using Trizol reagent (Invitrogen, USA) according to the manufacturer’s instructions. Trizol prepared RNA was reversely transcribed into cDNA using Superscript III reverse transcriptase (Invitrogen, USA) and random primers. Based on SYBR Green PCR Master Mix (Applied TaKaRa, Japan), qRT-PCR was subsequently performed on ABI PRISM 7300HT Sequence Detection System (Applied Biosystems, USA) using cDNA as template. 18s was used as a control for normalization. Primer sequences were listed in Supplementary Table 3.

**Western Blotting**

Cell lysates were prepared with cell lysis buffer (Beyotime Biotechnology, China). Protein concentrations were measured using a BCA assay (Thermo Scientific, USA). Total cell protein (30 μg) was subjected to SDS-polyacrylamide gel electrophoresis and transferred to PVDF membranes (Millipore, USA). After blocking in 5% non-fat milk in TBST for 1 h, the membranes were incubated with anti-SVCT-2 (A6740, Abclonal Technology, China), CD133 (A0219, Abclonal Technology, China), Oct-4 ([2750](https://www.cst-c.com.cn/products/primary-antibodies/oct-4-antibody/2750?N=4294956287&Ntt=oct4&fromPage=plp), CST, USA), phospo-H2AX (Ser139) ([9718](https://www.cst-c.com.cn/products/primary-antibodies/phospho-histone-h2a-x-ser139-20e3-rabbit-mab/9718?N=4294956287&Ntt=h2ax&fromPage=plp), CST, USA), p21 ([2947](https://www.cst-c.com.cn/products/primary-antibodies/p21-waf1-cip1-12d1-rabbit-mab/2947?N=4294956287&Ntt=p21&fromPage=plp), CST, USA), cleaved-PARP ([5625](https://www.cst-c.com.cn/products/primary-antibodies/cleaved-parp-asp214-d64e10-xp-rabbit-mab/5625?N=4294956287&Ntt=cleaved-parp&fromPage=plp), CST, USA), cleaved caspase-3 ([9661](https://www.cst-c.com.cn/products/primary-antibodies/cleaved-caspase-3-asp175-antibody/9661?N=4294956287&Ntt=cleaved+caspase-3&fromPage=plp), CST, USA), LC3B ([3868](https://www.cst-c.com.cn/products/primary-antibodies/lc3b-d11-xp-rabbit-mab/3868?N=4294956287&Ntt=lc3b&fromPage=plp), CST, USA), Beclin-1 ([4122](https://www.cst-c.com.cn/products/primary-antibodies/beclin-1-2a4-mouse-mab/4122?N=4294956287&Ntt=beclin-1&fromPage=plp), CST, USA), Lin28 (ab46020, Abcam, UK), β-actin ([3700](https://www.cst-c.com.cn/products/primary-antibodies/b-actin-8h10d10-mouse-mab/3700?N=4294956287&Ntt=Î²-actin&fromPage=plp), CST, USA). After incubation with fluorescein-conjugated secondary antibody, the blots were detected using an Odyssey fluorescence scanner (Li-Cor, Lincoln, NE, USA). The uncropped scans of western blots from the main figures are displayed in Supplementary Fig. 5.

**RNA Interference**

The Lentivirus-mediated short hairpin RNA (shRNA) expressing vector was purchased from Genechem Technology (Shanghai, China). The sequences of shRNA targeting SVCT-2 (shSVCT-2-1) were: sense: 5′-CCUCUCCCGAUUUAUAAAUdTdT-3′; antisense: 5′-AUUUAUAAAUCGGGAGAGGdTdT-3′; shSVCT-2-2: sense: 5′-GAAUCGAUCAAGUG UUGAAdTdT-3′; antisense: 5′-UUCAACACUUGAUCGAUUCdTdT-3′. Cells were seeded at ~50% confluence and [transfected](http://topics.sciencedirect.com/topics/page/Transfection) with optimal dilutions of lentivirus particles and polybrene. 48 h after [transfection](http://topics.sciencedirect.com/topics/page/Transfection), the cells were cultured in DMEM containing puromycin (2 μg/ml) to obtain the stable transfected cells.

**Intracellular VC and ROS measurement**

Total VC quantification was performed using the Ascorbic Acid Colorimetric Assay Kit (Biovision, CA). To measure intracellular ROS, cells were incubated in medium containing 2 mM VC for 30 min at 37°C. Then, 10 μM 2’,7’-Dichlorofluorescein diacetate (DCF-DA, Sigma) was added to the medium and cells were further incubated for 30 min. Cells were washed with phosphate-buffered saline (PBS), trypsinized, resuspendend with PBS and analyzed with a Moflo XDP flow cytometer (Beckman Coulter, USA).

**ATP detection**

The level of ATP was determined by the luciferinluciferase method using the ATP detection kit (Beyotime Biotechnology, China). Briefly, cells were lysed with a lysis buffer, followed by centrifugation at 12,000 × g for 5 min at 4°C. Then, 20 μl supernatant of samples mixed with 100 μl luciferase reagent for ATP detection using a microplate luminometer. The luminescence was linearly related to the ATP concentration and measured and the relative ATP level=ATP value/protein value.

**Cell cycle analysis**

Cell cycle profiles were determined by Cell Cycle and Apoptosis Analysis Kit (Beyotime Biotechnology, China). Cells were harvested by trypsinisation, fixed in cold 70% ethanol for 6~8 h, and incubated with propidium jodide (PI) and RNase for 30 min at room temperature. DNA distribution was analyzed with a Moflo XDP flow cytometer (Beckman Coulter, USA).

**Cell apoptosis measurement**

Cell apoptosis was evaluated by Annexin V-FITC apoptosis detection kit (Beyotime Biotechnology**,** China). After indicated treatment, HCC cells were harvested and stained according to manufacturer’s protocol. Data acquisition and analysis were performed in a Moflo XDP flow cytometer (Beckman Coulter, USA) and calculated by CellQuest software.

**Flow cytometry analysis**

HCC cells were incubated with APC-conjugated anti-EpCAM (Miltenyi Biotec, Germany) or PE-conjugated anti-CD133 (Miltenyi Biotec, Germany) followed by flow cytometry analysis using a Moflo XDP flow cytometer (Beckman Coulter, USA).

**Magnetic sorting**

HCC cell lines and human HCC primary cells were labeled with primary antibody for CD133 (Miltenyi Biotec, Germany) or OV6 (mouse IgG1, R&D Systems, USA). These cells were sorted on MACS LS columns (Miltenyi Biotec, Germany) followed by incubation with goat anti-mouse IgG microbeads (Miltenyi Biotec, Germany) magnetically. All the procedures were performed according to manufacturer’s instructions.

**Sphere formation**

800 or 3,000 cells were seeded in 24 or 6-well ultra-low attachment culture plates (Corning, USA) in DMEM/F12 (Gibco, USA) supplemented with 1% FBS, 20 ng/mL bFGF and 20 ng/mL EGF for 2 weeks. The number of spheroids was counted under inverted microscope (Olympus, Japan) and representative views were pictured.

**Histopathologic and immunohistochemical evaluation**

All sections were evaluated by two experienced pathologists who were blind to the clinical data of the patients. Based on the immunohistochemical analysis, the staining intensity of tumor cells was scored as 0 (negative staining), 1 (weak staining), 2 (moderate staining), and 3 (strong staining). Then the patients were subdivided into two groups: high SVCT-2 expression group (negative or weak staining) and low SVCT-2 expression group (moderate or strong staining).

**Supplementary Figures**


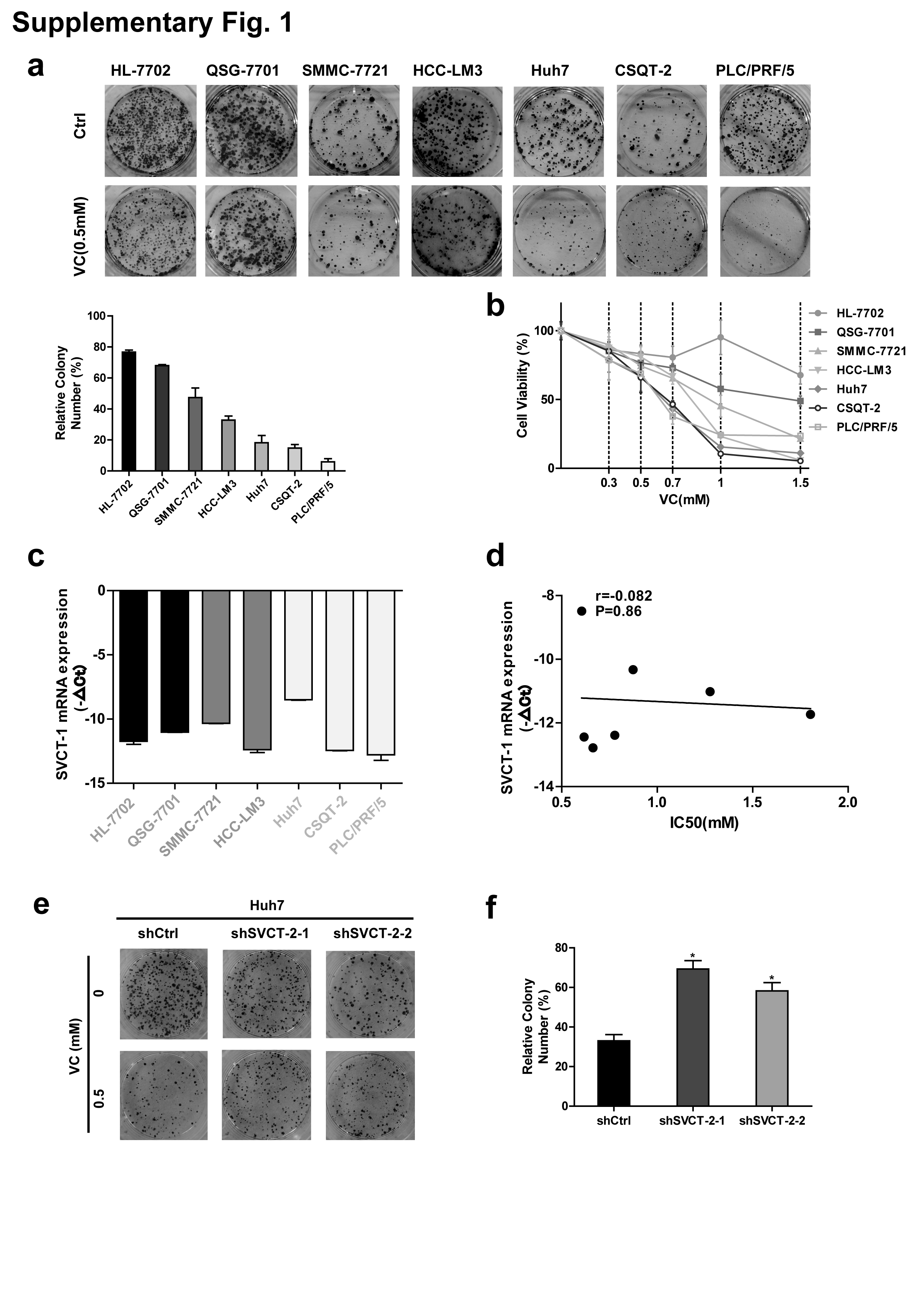


**Supplementary Fig. 1** SVCT-2, but not SVCT-1 controls the differential sensitivity to the cytotoxicity of VC. **a** Five HCC cell lines (SMMC-7721, HCC-LM3, Huh7, CSQT-2, and PLC/PRF/5) and two relative normal liver cells (HL-7702 and QSG-7701) were treated with 0.5 mM VC. Colony formation assay on the indicated cells was performed and stained using crystal violet after 7 days. Top: representative images of the colonies. Bottom: quantification of colonies in the same experiment. **b** Five HCC cell lines and two relative normal liver cells were treated with indicated concentrations of VC for 48 h. Cell viability was determined by the CCK-8 assay. **c** SVCT-1 mRNA expressions in HCC cell lines and relative normal liver cells were detected by qRT-PCR. **d** Correlation between SVCT-1 mRNA expression and IC50 values of VC in HCC cell lines and relative normal liver cells. **e**, **f** Huh7 cells transfected with SVCT-2-shRNA or scramble shRNA were treated with 0.5 mM VC. Colony formation assay on the indicated cells was performed and stained using crystal violet after 7 days. Representative images of the colonies (**e**). Quantification of colonies in the same experiment (**f**). Data are representative of at least three independent experiments and shown as mean ± s.d. (*, p < 0.05; **, p < 0.01; ***, p < 0.001).


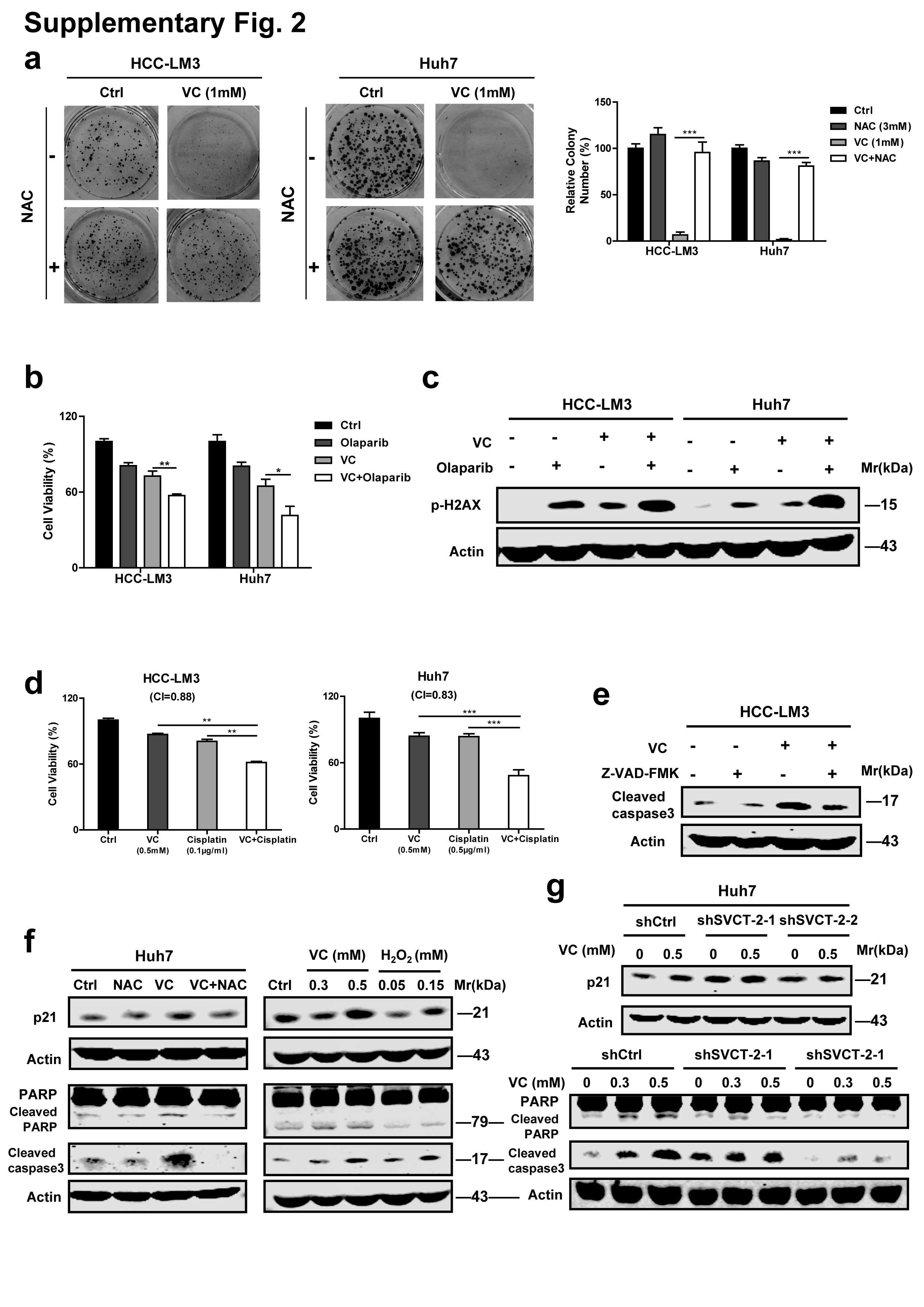


**Supplementary Fig. 2** SVCT-2-dependent mechanisms of pharmacological VC-induced cell death. **a** HCC cells were treated with 1 mM VC after pretreatment with 2 mM NAC. Colony formation assay on the indicated cells was performed and stained using crystal violet after 7 days. Left: representative images of the colonies. Right: quantification of colonies in the same experiment. **b** HCC cells were treated with 0.5 mM VC in the presence of the PARP inhibitor olaparib for 48h. Cell viability was analyzed by CCK-8 assay. **c** Western blot analysis showing expressions of p-H2AX in the indicated cells exposed to 0.5 mM VC and olaparib either alone or in combination for 48h. Samples derived from the same experiment and gels/blots were processed in parallel. **d** HCC cells were exposed to VC and cisplatin either alone or in combination at indicated concentration for 48h. Cell viability was determined by the CCK-8 assay. Combination index (CI) was calculated by CalcuSyn 2.1 software (Biosoft). CI < 1, CI = 1, and CI > 1 suggest synergism, additive effect, and antagonism, respectively. **e** Western blot analysis showing expressions of cleaved caspase3 in HCC-LM3 cells exposed to 0.5 mM VC with or without Z-VAD-FAM for 48h. Samples derived from the same experiment and gels/blots were processed in parallel. **f** Western blot analysis showing expressions of p21, cleaved PARP, and cleaved caspase3 in Huh7 cells exposed to 0.5 mM VC with or without NAC for 48h and p21, cleaved PARP, and cleaved caspase3 induced by VC or H_2_O_2_ in a dose dependent manner. Samples derived from the same experiment and gels/blots were processed in parallel. **g** Western blot analysis showing p21, cleaved PARP, and cleaved caspase3 expression in shSVCT-2 cells and shCtrl cells treated with VC for 48h. Samples derived from the same experiment and gels/blots were processed in parallel. Data are representative of at least three independent experiments and shown as mean ± s.d. (*, p < 0.05; **, p < 0.01; ***, p < 0.001).


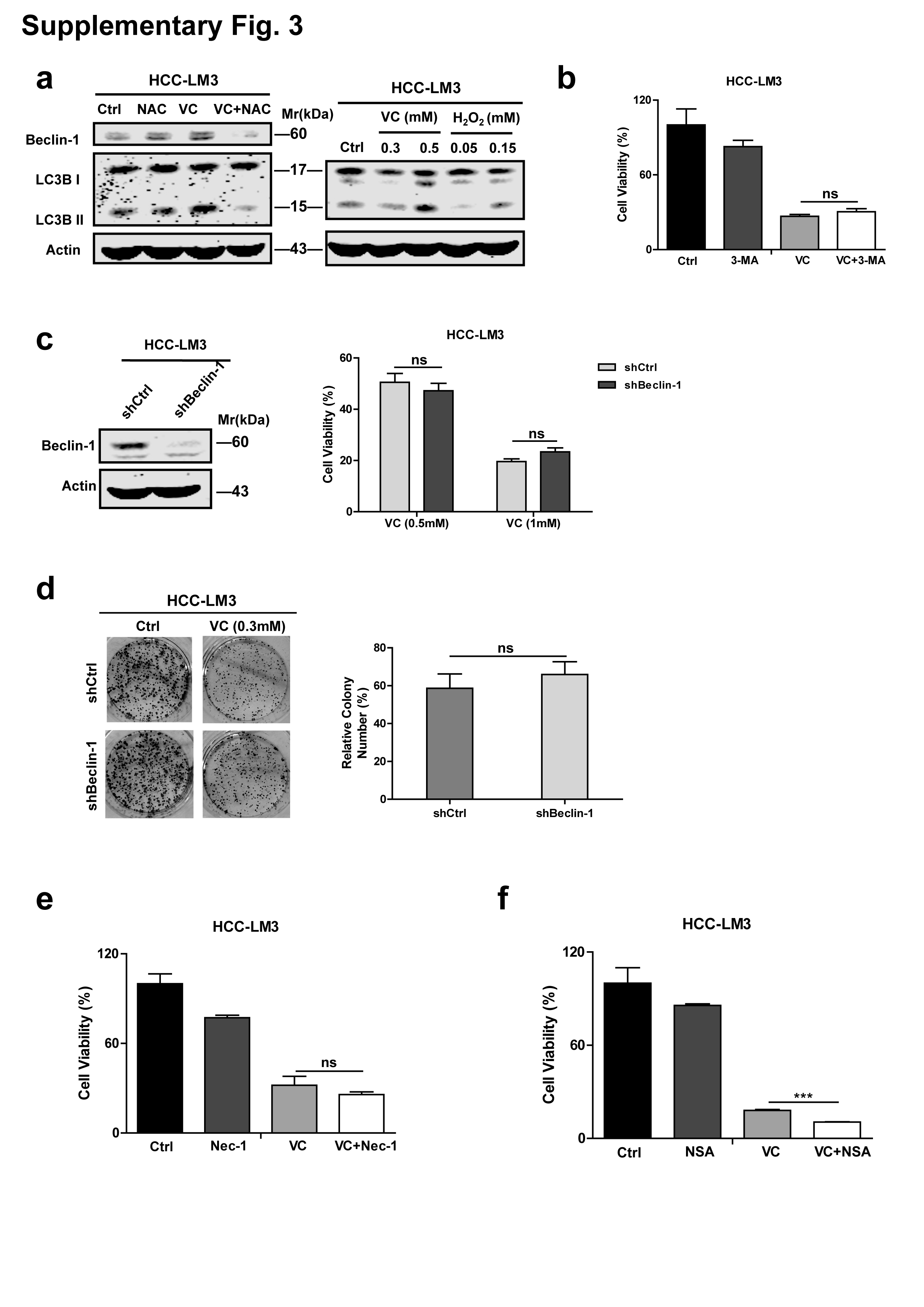


**Supplementary Fig. 3** Pharmacological VC induces cell death not through autophagy or necroptosis. **a** Left: western blot analysis showing expressions of Beclin-1 and LC3B II in HCC-LM3 cells exposed to 0.5 mM VC with or without NAC for 48h. Right: western blot analysis showing increase in LC3B II and decrease in LC3B I induced by VC or H_2_O_2_ in a dose dependent manner. Actin served as a loading control. Samples derived from the same experiment and gels/blots were processed in parallel. **b** HCC cells were treated with 1 mM VC for 48h after pretreatment with 5 mM 3-MA. Cell viability was determined by the CCK-8 assay. **c** Left: HCC-LM3 cells were transfected with Beclin-1-shRNA or scramble shRNA and the Beclin-1 expression was analyzed by immunoblotting. Samples derived from the same experiment and gels/blots were processed in parallel. Right: HCC-LM3 cells transfected with Beclin-1-shRNA or scramble shRNA were treated with indicated doses of VC for 48 h. Cell viability was determined by the CCK-8 assay. **d** Colony formation assay on the indicated cells was performed and stained using crystal violet after 7 days. Right: representative images of the colonies. Left: quantification of colonies in the same experiment. **e**, **f** HCC-LM3 cells were treated with 1 mM VC for 48h after pretreatment with necrostatin-1 (Nec-1) (**e**) or necrosulfonamide (NSA) (**f**). Cell viability was determined by the CCK-8 assay. Data are representative of at least three independent experiments and shown as mean ± s.d. (ns, no significance; *, p < 0.05; **, p < 0.01; ***, p < 0.001).


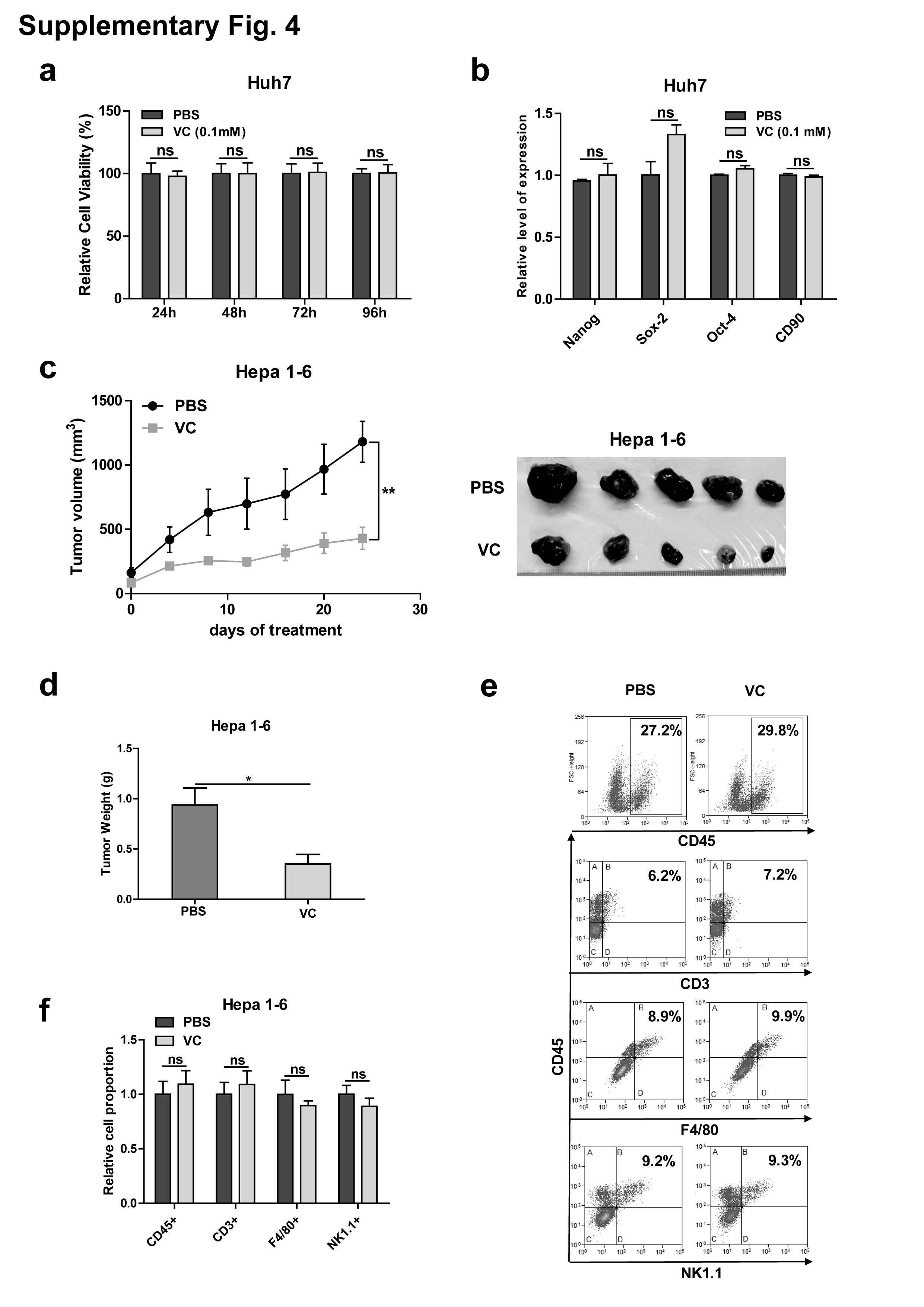


**Supplementary Fig. 4** Physiological concentration of VC does not promote HCC *in vitro* and pharmacological VC is not toxic to immune cells *in vivo*. **a** Huh7 cells were untreated or treated with 0.1 mM VC for 48 h. Cell viability was determined by the CCK-8 assay. **b** qRT-PCR analysis for stemness markers in HCC cells untreated or treated with 0.1 mM VC for 48h. **c**, **d** Relative growths (**c**) and weights (**d**) of tumors derived from Hepa1-6 cells subcutaneously inoculated into C57BL/6 mice after VC (4.0 g/kg) or vehicle (PBS) treatment. **e** Flow cytometric analysis for the proportion of immune cells (CD45+) and major immune cell subpopulations including T-lymphocytes (CD3+), macrophages (F4/80+), and natural killer (NK) cells (NK1.1+) in tumors from C57BL/6 mice after VC (4.0 g/kg) or vehicle (PBS) treatment. **f** Quantification of immune cell proportions in the same experiment. Data are representative of at least three independent experiments and shown as mean ± s.d. (ns, no significance; *, p < 0.05; **, p < 0.01; ***, p < 0.001).


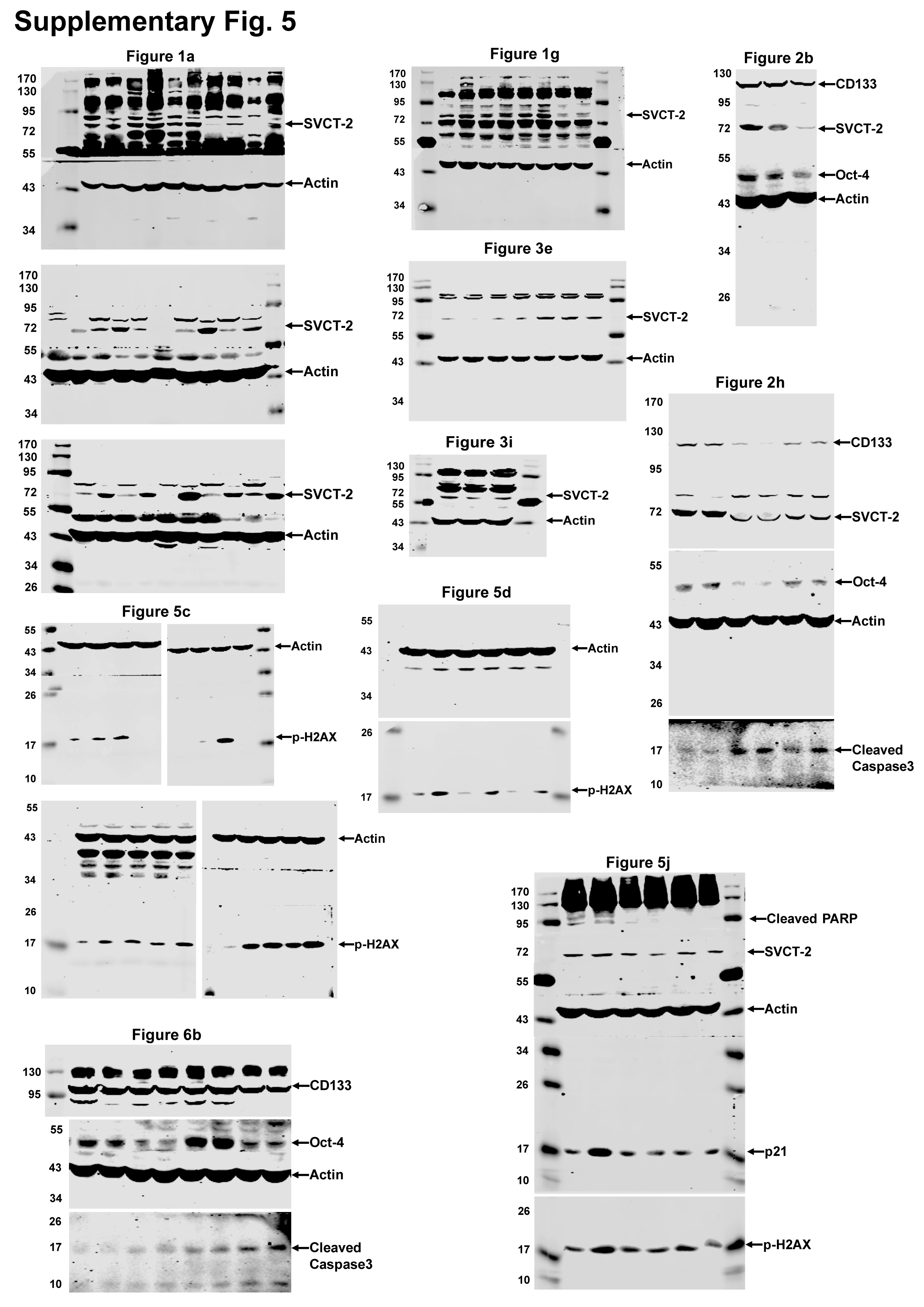


**Supplementary Fig. 5** The uncropped scans of western blots from the main figures.

**Supplementary Table 1 Clinicopathological characteristics of 104 HCC cases**

| **Characteristic** | **SVCT-2 expression (number, %)** | | **P value** |
| --- | --- | --- | --- |
|  | **Low (n=43)** | **High (n=61)** |  |
| **Age** |  |  | 0.938 |
| <60 | 25 (58.1%) | 35 (57.4%) |  |
| ≥60 | 18 (41.9%) | 26 (42.6%) |  |
| **Gender** |  |  | 0.723 |
| Male | 39 (90.7%) | 54 (88.5%) |  |
| Female | 4 (9.3%) | 7 (11.5%) |  |
| **HBV infection** |  |  | 0.723 |
| Yes | 39 (90.7%) | 54 (88.5%) |  |
| No | 4 (9.3%) | 7 (11.5%) |  |
| **Tumor size** |  |  | 0.028* |
| <5cm | 16 (37.2%) | 11 (18.0%) |  |
| ≥5cm | 27 (62.8%) | 50 (82.0%) |  |
| **Tumor number** |  |  | 0.043* |
| Single | 27 (62.8%) | 26 (42.6%) |  |
| Multiple | 16 (37.2%) | 35 (57.4%) |  |
| **AFP** (μg/L) |  |  | 0.997 |
| <20 | 12 (27.9%) | 17 (27.9%) |  |
| ≥20 | 31 (72.1%) | 44 (72.1%) |  |
| <400 | 19 (44.2%) | 25 (41.0%) | 0.745 |
| ≥400 | 24 (55.8%) | 36 (59.0%) |  |
| **Tumor differentiation** |  |  | 0.971 |
| Ⅰ-Ⅱ | 10 (23.3%) | 14 (23.0%) |  |
| Ⅲ-Ⅳ | 33 (76.7%) | 47 (77.0%) |  |
| **Liver cirrhosis** |  |  | 0.410 |
| Yes | 27 (62.8%) | 43 (70.5%) |  |
| No | 16 (37.2%) | 18 (29.5%) |  |
| **Microscopic tumor thrombus** |  |  | 0.874 |
| Absent | 19 (44.2%) | 26 (42.6%) |  |
| Present | 24 (55.8%) | 35 (57.4%) |  |
| **Macroscopic tumor thrombus** |  |  | 0.043* |
| Absent | 37 (86.0%) | 42 (68.9%) |  |
| Present | 6 (14.0%) | 19 (31.1%) |  |
| **Metastasis** |  |  | 0.212 |
| No | 39 (90.7%) | 50 (82%) |  |
| Yes | 4 (9.3%) | 11 (18%) |  |

*, p < 0.05.

**Supplementary Table 2 Clinicopathological characteristics of 613 HCC cases**

| **Characteristic** | **Number (%)** | | **P value** |
| --- | --- | --- | --- |
|  | **VC non-use**  **(n=274)** | **VC use**  **(n=339)** |  |
| **Age** |  |  | 0.134 |
| <60 | 193 (70.4%) | 257 (75.8%) |  |
| ≥60 | 81 (29.6%) | 82 (24.2%) |  |
| **Gender** |  |  | 0.722 |
| Male | 234 (85.4%) | 286 (84.4%) |  |
| Female | 40 (14.6%) | 53 (15.6%) |  |
| **HBV infection** |  |  | 0.767 |
| Yes | 228 (83.2%) | 279 (82.3%) |  |
| No | 46 (16.8%) | 60 (17.7%) |  |
| **Tumor size** |  |  | 0.686 |
| <5cm | 120 (43.8%) | 154 (45.4%) |  |
| ≥5cm | 154 (56.2%) | 185 (54.6) |  |
| **Tumor number** |  |  | 0.387 |
| Single | 230 (83.9%) | 293 (86.4%) |  |
| Multiple | 44 (16.1%) | 46 (13.6%) |  |
| **AFP** (μg/L) |  |  | 0.212 |
| <20 | 122 (44.5%) | 134 (39.5%) |  |
| ≥20 | 152 (55.5%) | 205 (60.5%) |  |
| <400 | 117 (64.6%) | 155 (63.3%) | 0.770 |
| ≥400 | 64 (35.4%) | 90 (36.7%) |  |
| **Tumor differentiation** |  |  | 0.172 |
| Ⅰ-Ⅱ | 159 (58.0%) | 178 (52.5%) |  |
| Ⅲ-Ⅳ | 115 (42.0%) | 161 (47.5%) |  |
| **Liver cirrhosis** |  |  | 0.129 |
| Yes | 182 (66.4%) | 205 (60.5%) |  |
| No | 92 (33.6%) | 134 (39.5%) |  |
| **Microscopic tumor thrombus** |  |  | 0.441 |
| Absent | 120 (43.8%) | 138 (40.7%) |  |
| Present | 154 (56.2%) | 201 (59.3%) |  |
| **Macroscopic tumor thrombus** |  |  | 0.605 |
| Absent | 243 (88.7%) | 296 (87.3%) |  |
| Present | 31 (11.3%) | 43 (12.7%) |  |
| **Tumor encapsulation** |  |  | 0.115 |
| Incomplete | 132 (48.2%) | 185 (54.6%) |  |
| Complete | 142 (51.8%) | 154 (45.4%) |  |

**Supplementary Table 3** Primer List

| **Gene** | **Forward primer (5’-3’)** | **Reverse primer (5’-3’)** |
| --- | --- | --- |
| *SVCT-1* | GCTACCCACAGAGCCTAAGTT | TGACTAACCATGTGCTGGTCG |
| *SVCT-2* | CTTCACTCTTCCGGTGGTGAT | TTTCCGTAGTGTAGATCGCCA |
| *CD133* | TTACGGCACTCTTCACCT | TATTCCACAAGCAGCAAA |
| *EpCAM* | TCGCGTTCGGGCTTCTGCTT | GGGCCCCTTCAGGTTTTGCT |
| *Nanog* | CATGAGTGTGGATCCAGCTTG | CCTGAATAAGCAGATCCATGG |
| *Sox-2* | CAAGATGCACAACTCGGAGA | GCTTAGCCTCGTCGATGAAC |
| *Oct-4* | AGTGAGAGGCAACCTGGAGA | ACACTCGGACCACATCCTTC |
| *18S* | CGGCTACCACATCCAAGGAA | GCTGGAATTACCGCGGCT |
